# Combining Cancer Vaccines with Immunotherapy: Establishing a New Immunological Approach

**DOI:** 10.3390/ijms22158035

**Published:** 2021-07-27

**Authors:** Chang-Gon Kim, Yun-Beom Sang, Ji-Hyun Lee, Hong-Jae Chon

**Affiliations:** 1 Yonsei Cancer Center, Department of Internal Medicine, Division of Medical Oncology, Yonsei University College of Medicine, Seoul 03722, Korea; inspector@yuhs.ac (C.-G.K.); jhlee0811@yuhs.ac (J.-H.L.); 2 CHA Bundang Medical Center, Medical Oncology, CHA University School of Medicine, Seongnam 13497, Korea; ybsang85@chamc.co.kr

**Keywords:** therapeutic cancer vaccine, combination immunotherapy, immune checkpoint inhibitor, tumor microenvironment

## Abstract

Therapeutic cancer vaccines have become increasingly qualified for use in personalized cancer immunotherapy. A deeper understanding of tumor immunology and novel antigen delivery technologies has assisted in optimizing vaccine design. Therapeutic cancer vaccines aim to establish long-lasting immunological memory against tumor cells, thereby leading to effective tumor regression and minimizing non-specific or adverse events. However, due to several resistance mechanisms, significant challenges remain to be solved in order to achieve these goals. In this review, we describe our current understanding with respect to the use of the antigen repertoire in vaccine platform development. We also summarize various intrinsic and extrinsic resistance mechanisms behind the failure of cancer vaccine development in the past. Finally, we suggest a strategy that combines immune checkpoint inhibitors to enhance the efficacy of cancer vaccines.

## 1. Introduction

Since the approval of sipuleucel-T by the U.S. Food and Drug Administration (FDA) in 2010, impressive progress has been made in the field of therapeutic cancer vaccine development due to the advancement in tumor immunology and the introduction of immune checkpoint inhibitors (ICIs) [1,2,3,4,5,6,7,8,9]. Therapeutic cancer vaccines primarily aim to induce an adaptive immune response against tumor antigens, leading to tumor regression [10,11]. The efficacy of cancer vaccines generally depends on four factors: the optimized delivery of dendritic cells (DCs) that successfully take up and present the tumor antigens, the activation of effector T cells, trafficking activated T cells into the tumor microenvironment (TME), and the induction of a durable memory response [12,13,14,15,16,17,18]. All these requirements can be achieved by activating DCs to an optimal level with adjuvants [19], directly loading tumor antigens into autologous DCs [20], and reinvigorating exhausted T cells by ICIs [21]. In this review, we discuss the combination immunotherapies that enhance the efficacy of cancer vaccines. We also describe the mechanisms of resistance that pose significant challenges and how to overcome these challenges through the use of immunotherapy.

## 2. Target Antigens for Therapeutic Cancer Vaccine

Traditionally, tumor-associated antigens (TAAs) have been regarded as targets for therapeutic cancer vaccines (Table 1) [22]. TAAs are self-antigens expressed selectively on tumor cells [23]. Various types of TAAs, including differentiation antigens [24], cancer testis antigens [25], and overexpressed antigens [26], have frequently been employed for vaccination strategies [27]. However, the existence of central tolerance hinders the antitumor response of high-affinity T cells against TAAs [28].

On the other hand, tumor-specific antigens (TSAs) include oncoviral antigens, private neoantigens, and shared neoantigens [29,30]. TSAs are well recognized by high-affinity T cells and are not largely influenced by central tolerance. Moreover, TSAs do not induce autoimmune disorders [31]. Oncoviral antigens, such as E6 and E7 of the human papillomavirus (HPV) [32], are non-self antigens [33] and can act as tumor antigens. Neoantigens are generated by nonsynonymous somatic mutations or frame shift mutations [34,35] in tumor cells without an intact mismatch repair system [36,37]. Tumor mutation burden (TMB) is determined by the amount of gene mutations within cancer cells and shows a strong correlation with the effectiveness of neoantigen-based vaccine [38,39]. Based on this rationale, patients with high TMB can be good candidates for private neoantigen-based vaccine therapy. In contrast, patients with low TMB can be treated better with vaccines based on TAAs [10]. Collectively, the selection of proper tumor antigens for individual patients is one of the factors determining success of therapeutic cancer vaccines.

## 3. Platform of Cancer Vaccine

Platforms of cancer vaccines are often divided into cellular, viral vector, or molecular (DNA, RNA, or peptide) vaccines [40]. Cellular vaccines are made from autologous tumor cells obtained from patients or cells that are derived from allogeneic tumor cell lines [41]. DCs are the best candidate for developing cellular cancer vaccines due to their ability to engulf, process, and present tumor antigens [42]. Viral vector vaccines can enhance antitumor immune responses resulting from the delivery of tumor antigens that effectively prime T cells [43]. Moreover, viral vaccines can become oncolytic by genetically engineering them to selectively infect tumor cells, disseminate, and eradicate tumor cells [44].

Vaccines based on molecular platforms have been widely investigated due to their ease of manufacture. DNA vaccines are relatively easier to develop, combine with adjuvants, and are able to induce enriched expression of tumor antigens. However, in order to accomplish these, the vaccines must be processed by additional mechanisms including transcription, translation, and cross-presentation [45,46]. DNA vaccines consist of closed circular DNA plasmids, often termed as naked DNA; these encode target tumor antigens and immunomodulatory molecules for T cell education and specific recognition of tumor cells [47,48]. To optimize T-cell responses against tumors, a relatively high dosage of DNA vaccines must be intramuscularly injected, and then its delivery must be enhanced by electroporation [49]. DNA vaccines constructed with multiple synthetic neoantigens have been reported to induce cytotoxic T-cell responses against the tumor in preclinical models [50]. Furthermore, the development of fusion DNA products that consist of selective antigens and chemokines for the recruitment of DCs or T cells has drawn attention in the field of therapeutic DNA cancer vaccines [51,52,53].

RNA vaccines do not require transcription to induce antigen expression [54]. RNA vaccines are constructed in vitro to encode antigens and present proteins after internalization to stimulate immune responses [55]. RNA vaccines are able to produce large amounts of antigens and co-stimulatory signals with reduced risks of infection or insertional mutagenesis, and their manufacture is relatively simple and cost-effective [56]. However, RNA vaccines often face challenges with poor stability and inefficient delivery [57]. RNA vaccines can be directly delivered into the lymph nodes or injected intravenously after encapsulation with nanocarrier particles called lipoplex nanoparticles [58]. Recently, the delivery of mRNA via lipoplex nanoparticles containing four shared melanoma tumor antigens in combination with a PD-1 inhibitor has been shown to establish a robust T cell response, leading to a reduced volume of the tumor [59], indicating that a feasible combination strategy can achieve improved efficacy of the RNA vaccine platform.

Peptide-based cancer vaccines were developed based on the interaction between the T cell receptor (TCR) and the peptide–MHC complex [60]. Initial application of peptide-based cancer vaccines was performed with accurate MHC-I binding short peptides [61]. However, short peptides derived from the vaccine bind to MHC-I molecules in all nuclear cells and are highly likely to be rapidly degraded, resulting in a suboptimal T cell response and consequently impaired treatment response [62]. To overcome such issues, vaccines that use synthetic long peptide (SLP) were developed [63]. SLP vaccines are considered highly immunogenic because they employ DC-mediated antigen presentation for activation of both CD8^+^ T effector cells and CD4^+^ T helper cells [64]. Both CD4^+^ and CD8^+^ T cell responses were shown to be vigorously induced by SLP vaccines designed to induce a T cell response against NY-ESO-1 with incomplete Freund’s adjuvant [65]. Moreover, SLP vaccination against E6 and E7 viral oncoproteins resulted in significant clinical efficacy to a level similar to monotherapy [66]. Overall, the mechanisms underlying the effective delivery of peptides at the correct site warrant further investigation for future cancer vaccine design.

## 4. Resistance Mechanisms Compromising Vaccine Efficacy

**Figure 1:**
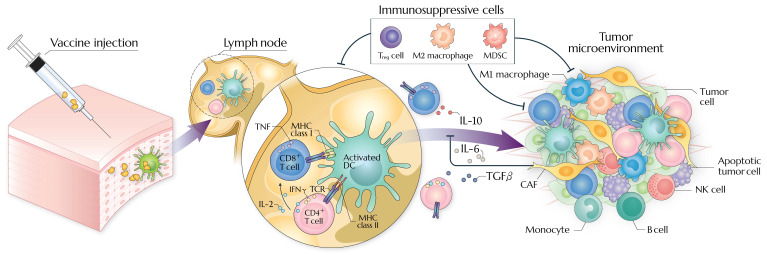
Modes of action and resistance mechanisms of therapeutic cancer vaccine. At the vaccine site, dendritic cells take up antigen and move to draining lymph nodes to prime T cells. In the tumor microenvironment, M2 macrophages, myeloid-derived suppressor cells, regulatory T cells, and cancer-associated fibroblast mainly impair activation of tumor-reactive T cells and fate decision of monocyte to M1 macrophage. Soluble factors (e.g., IL-6, IL-10, and TGF-β) block migration and activation of tumor-reactive T cells and NK cells. MDSCs, myeloid-derived suppressor cells; CAFs, cancer-associated fibroblasts; Treg, regulatory T cells.

Tumor cells evade vaccine-induced antitumor immunity in an intrinsic or extrinsic manner, establishing immunosuppressive TME (detailed in Figure 1) [67,68,69]. T-cell-based cancer immunotherapy is hampered by insufficient immunogenicity, high tumor burden, and exclusion of T cells within the TME [70,71,72]. Moreover, antitumor immune responses are difficult to be elicited in tumors harboring specific genetic mutations [73] or molecular profiles [74].

Chronologically, resistance to immunotherapy, including therapeutic vaccines, may arise from a lack of direct response to treatment (primary resistance) or initial response followed by progression (acquired resistance) [2]. There are numerous underlying causes that accelerate such immune escape [75]. Mechanisms of resistance are often described as being either tumor-intrinsic (a property of the tumor cell itself) [76], or tumor-extrinsic (stromal components of the tumor) [77,78,79,80,81]. Additionally, it is crucial to note the fact that the same mechanisms that initially enhanced responsiveness to immunotherapy could also lead to the emergence of acquired resistance, as has been reported in the case of interferon signaling pathways [82].

### 4.1. Tumor-Intrinsic Resistance

Tumor-intrinsic resistance that hinders the efficacy of immunotherapies, including ICIs, adoptive cell transfer, and therapeutic vaccines, can be attributed to insufficient tumor antigen expression [83], modified antigen processing pathways [84], and the depletion of human leukocyte antigen (HLA) expression linked to a diminished display of tumor antigens [85], all of which interfere with tumor cell recognition by T cells. For example, resistance against therapeutic vaccination that delivers autologous tumor cells and Bacillus Calmette–Guérin (BCG) in patients with melanoma can be driven by the loss of HLA class I, which can also be observed in the administration of autologous virus-specific T cells for Merkel cell carcinoma and vaccination with BCG for bladder cancer [86]. Resistance against IFN-γ signaling inhibits the rapid antitumor response induced by immunotherapy-derived T cell activation [87]. Constitutive expression of PD-L1 or other ligands for immune checkpoint molecules often restrain the effector functions as well as activation of T cells [88]. Furthermore, elevated expression of various immune checkpoint molecules on neoantigen-specific T cells is significantly associated with unresponsiveness to the combined treatment of neoantigen vaccine and ICIs in patients with bladder cancer, melanoma, and non-small cell lung cancer (NSCLC) [89].

Gene expression analysis of samples derived from humans and mice after ICI administration revealed that tumor endogenous WNT-β-catenin signaling or PTEN upregulated expression of immunosuppressive cytokines, downregulated expression of chemokines that are important for recruiting effector T cells, and resulted in the loss of DCs and CD8^+^ T cells in the TME [90]. Upregulated expression of β-catenin provokes T cell exclusion and suppresses the infiltration of activated T cells following vaccination, subsequently causing acquired resistance to vaccination with IL-12 [91]. Therapeutic vaccines have been revealed to establish T cell infiltration into tumors; however, T cells often fail to infiltrate the immune-excluded lesions [92]. Furthermore, bone marrow-derived effector or suppressive cells tend to participate in primary and acquired resistance to therapeutic vaccination despite vigorous vaccine-mediated infiltration of T cells in mice and patients [93]. This is partially related with the fact that programmed cell death driven by T cells was not completely linked to programmed cell elimination.

### 4.2. Tumor-Extrinsic Resistance

Tumor-extrinsic resistance that leads to diminished immune response is often induced due to systemically and/or locally accumulated immunosuppressive cells including regulatory T cells (Tregs) [94], myeloid-derived suppressor cells (MDSCs) [95], tumor-associated macrophages (TAMs) expressing tumor-promoting phenotype [96], and cancer-associated fibroblasts [97]. These cells have been shown to suppress the activation, proliferation and effector function of T cells via expression of inhibitory receptors and production of diverse immunosuppressive cytokines, arginase 1, inducible nitric oxide synthase, and reactive oxygen species [98,99,100,101]. Increased frequency of Tregs and MDSCs has been significantly correlated with the reduced efficacy of ICIs and establishment of primary resistance [102,103] and limited efficacy of antitumor T cell immunity induced by DC-based vaccination. Moreover, cancer-associated fibroblasts often exert mechanisms of resistance against vaccines as they actively reconstitute the extracellular matrix, inhibit proliferation and migration of DCs, restrain T cell infiltration, and form a dense fibrous matrix in which MDSCs accumulate [104].

Macrophages can be subdivided into either M1- or M2-like cells, depending on their pro- or anti-inflammatory roles, respectively [105]. The pro-tumorigenic M2-like macrophages comprise the predominant population of TAMs in the TME, whereas antitumorigenic M1-like macrophages exist as a less abundant subset of TAMs [106]. TAMs can be derived from circulating monocytes which migrate into the TME by a chemotactic gradient generated by soluble factors such as CCL2 and IL-1β or from tissue-resident macrophages by repolarization. Colony stimulating factor 1 (CSF1) is a key element involved in TAM differentiation from a progenitor lineage [107]. Apart from their immunosuppressive roles, under certain circumstances in TME, TAMs can contribute to the adaptive immune system and the direct killing of tumor cells [108]. Recently, it was reported that the extent of T cell responses induced by vaccines and therapeutic approaches in mice to promote tumor regression are highly dependent on the accumulation of macrophages and/or neutrophils [109,110]. However, our current understanding of cell engineering for improving the efficacy of vaccination is limited, especially due to the insufficient information regarding specific cell surface markers on TAMs subpopulations. Nevertheless, these obstacles are expected to be resolved as our knowledge expands, together with the development of technologies that can be used to recognize the specific cell types that most effectively provide clinical benefit. This will make a huge contribution to therapeutic pipeline development accompanied with enhanced benefit of cancer vaccines. Currently, there are only a small number of therapeutic cancer vaccines that demonstrate meaningful antitumor efficacy, which limits our understanding on how primary and acquired resistance mechanisms influence response to therapeutic cancer vaccines. Such complex mechanisms might be unveiled by studying the underlying responsiveness and resistance to therapeutic vaccination such as those targeting HPV-associated tumors. Previously, a significant correlation was found between the frequency of immunosuppressive myeloid cells and poor immunogenicity following administration of therapeutic HPV vaccines in cervical cancer patients [92]. Notably, recent studies have demonstrated that such resistance can be avoided by the deletion of suppressor cells [96,111].

Taken together, much work is still required to elucidate the various mechanisms underlying resistance to vaccination. As described above, identifying and understanding these resistance mechanisms in a comprehensive manner will optimize potential combinations.

## 5. Combination Therapies with ICIs

In the TME, therapeutic cancer vaccines and ICIs can complement each other. During cancer progression, expression of immune checkpoint molecules is progressively increased on effector T cells, leading to diminished cytotoxic killing activity against tumor cells. This phenomenon, termed “T cell exhaustion”, can be restored by a number of approaches. Recent breakthrough results from ICIs that reinvigorate T cell exhaustion have been applied to treat various types of cancer. Importantly, anti-PD-1, anti-PD-L1, and anti-CTLA-4 antibodies have contributed to the huge success in treating a considerable number of patients with various cancer types [71]. However, some patients either do not respond to such treatment or even experience hyperprogressive disease [95,112]. Such unresolved issues often arise from the heterogeneity of individual tumors that include mechanisms of resistance to vaccines or ICIs and the types of vaccine platform. Therefore, a combination of therapeutic approaches designed to tackle these challenges is needed to guarantee clinical impact in diverse cancer types.

### 5.1. Biological Rationale of Combinatory Strategies

Pre-existing tumor-specific T lymphocytes play an important role during cancer immunotherapy [113]. The administration of therapeutic cancer vaccine aims to enrich populations of tumor-recognizing T lymphocytes, which are further augmented by combination with ICIs. Accordingly, the treatment outcome of patients treated with PD-1 inhibitors who progressed after platinum-based chemotherapy for recurrent HPV-positive head and neck squamous cell carcinoma were associated with an enriched population of pre-existing HPV-recognizing T lymphocytes already present in the TME [114]. ICI monotherapy is a widely used treatment strategy, and treatment of ICI alone has shown a great deal of promise in treating several types of cancer including NSCLC and melanoma [115]. However, in the case of non-immunogenic tumors such as prostate and pancreatic cancers, ICI treatment alone failed to provide successful outcomes [116,117]. Mechanistically, therapeutic cancer vaccines establish and activate tumor-specific T cells in the periphery, which subsequently localize them to TME. Moreover, cancer vaccines-induced cell death leads to the release of diverse tumor antigens followed by subsequent strong immune responses, termed as antigen spreading [118]. During the activation of tumor-specific T cells, upregulation of immune checkpoint molecules is frequently observed [119]. Therefore, a combined treatment of ICIs and cancer vaccines can be a promising therapeutic approach that enhances antitumor immune responses.

The immunosuppressive environment established in the TME limits the effector functions of T cells despite activation of antitumor immune responses, thereby presenting a major challenge in achieving vaccine efficacy. For example, CTLA-4, which is often found in helper T cells and Tregs, interacts with its ligands CD80 or CD86, exerting inhibitory signals during antigen presentation in the periphery. Inhibitors targeting CTLA-4 have been shown to promote vaccine-induced tumor-specific T cell response directly by inhibiting such inhibitory signals [120]. In addition, the blockade of CTLA-4 increases effector T-cell-to-Treg ratios in the TME [121], inducing a shift in the intratumoral balance from an immunosuppressive to a permissive state. PD-1 is another representative co-inhibitory receptor that modulates the cytotoxic activity and proliferation of tumor-specific T cells by binding with PD-L1 or PD-L2 [122]. ICIs targeting PD-1 prevent vaccine-activated T cells to become senescent cells in the TME [123], thus establishing long-term antitumor T cell immune response and restoring production of cytokines required for controlling tumor growth. Overall, ICIs not only promote, but also sustain vaccine-induced immune responses by modulating suppressive characteristics found in the TME and blocking negative regulations of antitumor responses.

### 5.2. Preclinical Evidence for Combining ICIs and Vaccines

Several studies have attempted to reveal the mechanisms behind the synergistic benefits that could arise from combining ICIs with vaccines. Soares et al. revealed that administration of anti-PD-1 together with gene-transfected tumor cell (GVAX) vaccine resulted in increased survival rates and effective T cell response in murine models of pancreatic ductal adenocarcinoma [124], where treating with checkpoint inhibitor alone failed to provide clinical efficacy. Such synergistic effects from combined treatment were also reported in another non-immunogenic tumor model. Administration of vaccines containing DC tumor lysates followed by anti-PD-1 antibody treatment prolonged survival of mice with large-sized glioma tumors, whereas individual treatment alone did not provide antitumor efficacy [125]. Moreover, treatment with a PD-1 checkpoint inhibitor together with an adenovirus vaccine that targets the HPV-E6/E7 viral proteins resulted in enhanced antitumor response in a mouse model of HPV-associated tumor [126]. These findings highlight the synergistic impacts provided by the vaccine and ICI combination and suggest that inhibition of various types of checkpoints may synergistically enhance the vaccine efficacy.

The synergistic impact of a combination of anti-CTLA-4 antibodies and cancer vaccines has also been assessed in numerous preclinical studies. For instance, delivering GVAX in combination with anti-CTLA-4 antibody induced synergistic effects in controlling the tumor size and enhancing antitumor immune responses in melanoma and prostate cancer models [127]. Wada et al. described the significance of time when the combination of anti-CTLA-4 antibody and GM-CSF GVAX vaccine takes place in the Pro-TRAMP prostate cancer model, indicating that anti-CTLA-4 antibody should be given subsequent to vaccination to guarantee synergistical benefits [128]. This may suggest that administration of CTLA-4 blockade after vaccination could compromise compensatory expansion of Tregs, which could restrict the initiation of effective antitumor responses [129].

A combination treatment of anti-CTLA-4 and anti-PD-1 was shown to increase the number of activated T cells and the effector T-cell-to-Treg ratios in a murine model following vaccination [130]. Duraiswamy et al. demonstrated that a simultaneous blockade of both PD-1 and CTLA-4 in the presence of GVAX vaccine induced 100% rejection of CT26 colorectal tumors in mice and 75% in ID8-VEGF ovarian cancer [131]. Numerous efforts have been made to analyze the effects of blocking newly discovered targets including T cell immunoreceptor with immunoglobulin and immunoreceptor tyrosine-based inhibitory motif domains (TIGIT), lymphocyte activation gene 3 (LAG3), T cell immunoglobulin 3 (TIM3), V domain immunoglobulin inhibitor of T cell activation (VISTA), and B7-H3 [132,133]. Inducible co-stimulator (ICOS) has been recognized as a member of the CD28 family that belongs to costimulatory molecules responsible for T helper 2 (Th2) cell dysfunction and contextual cytokine responses [134]. Activation of ICOS by vaccines that induce expression of ICOS ligands was shown to provide synergistic efficacy when they were treated with CTLA-4 blocking antibodies in a preclinical study [135]. Likewise, upregulation of ICOS followed by the treatment of currently approved anti-CTLA-4 and anti-PD-1 antibodies led to improved outcomes with predictive clinical implications. Currently, research on merging novel immune checkpoint blockades and therapeutic cancer vaccines is actively underway.

### 5.3. Clinical Evidence for Combining ICIs and Vaccines

Talimogene laherparepvec (T-VEC) is a genetically modified vaccine that contains intralesional oncolytic viral proteins of herpes simplex virus type 1 (HSV-1), in which viral genes are partially deleted and substituted by GM-CSF gene [136]. The efficacy of T-VEC coupled with pembrolizumab treatment was evaluated as a phase Ib trial for the treatment of stage IIIB to IV unresectable melanoma. In this clinical trial, 21 patients received a dose of T-VEC (4 mL × 10^6^ pfu/mL), up to a total dose of 4 mL × 10^8^ pfu/mL every 2 weeks [137,138]. Intravenous administration of 200 mg of pembrolizumab was performed with subsequent delivery of T-VEC. This resulted in a 62% confirmed objective response rate, which was almost double that of the phase 3 clinical studies of T-VEC (26%) and pembrolizumab (34%), with a 33% complete response rate based on immune-related criteria for response. Patients who responded to this combination therapy showed increased lymphocyte infiltration, PD-L1 expression, and IFN-γ expression. The combination therapy did not have worse toxicity profiles compared with monotherapy, with the most commonly observed adverse effects including fatigue, chills, fever, rash, and arthralgia. Only one patient with combination-related grade 1 adverse event was hospitalized, whereas other grade 3–4 adverse events were overlapped with those previously documented in patients treated with pembrolizumab treatment alone. The systemic administration of pembrolizumab is currently being tested in an ongoing additional phase 3 KEYNOTE-034 trial along with intralesional injection of T-VECs (NCT02263508). In addition, a phase Ib study also evaluated the efficacy of pembrolizumab with T-VEC in 36 patients with advanced head and neck cancer [139]. An initial dose of T-VECs (8 mL × 10^6^ pfu/mL) was injected to intralesional area with subsequent doses (8 mL × 10^8^ pfu/mL) and treatment with pembrolizumab (200 mg) every 3 weeks. Preliminary data from this study reported a 16.7% objective response rate (six patients, including five patients with PD-L1-positive tumor) and 38.9% disease control rate (14 patients, including 11 patients with PD-L1-positive tumor). Symptoms including fever (36.1%), dyspnea (33.3%), and fatigue (25.0%) were the most common adverse events induced by the combination therapy. Grade 3 or 4 adverse reactions were experienced by 24 patients (66.7%), of which 2 (5.6%) and 1 (2.8%) patients on T-VEC and pembrolizumab treatment, respectively, terminated the trial.

In the case of peptide-based vaccines, the effect of combined treatment with nivolumab was evaluated in a phase 1 trial of patients with ipilimumab-naïve or -refractory advanced melanoma [140]. In this clinical trial, 90 participants with stage III to IV unresectable melanoma received a peptide vaccine and various doses of nivolumab (1 to 10 mg/kg with Montanide ISA 51 VG) in the presence or absence of peptide-based vaccination (gp100/MART-1/NY-ESO-1 with Montanide). In both ipilimumab-naïve and -refractory patients, the response rate was 25% and the durable response was observed for up to 140 weeks upon treatment with nivolumab and peptide vaccination. The combinatorial therapy appeared to be safe and well tolerated, without the incidence of treatment-related deaths. Adverse reactions such as fatigue and injection site reactions were commonly observed, but they were predominantly mild to moderate symptoms that could be easily managed. A range of symptoms including optic neuritis, fever, pneumonia and rash were detected as grade 3 immune-related adverse events (irAEs) that can be treated with the prednisone taper, as previously described for nivolumab. A phase I trial of nivolumab that was treated together with multipeptide vaccine as an adjuvant in resected stage IIIC to IV melanoma stages was performed by the same group [141]. In this study, the safety and efficacy of extended doses of nivolumab and peptide vaccine followed by nivolumab maintenance were tested in 33 patients. The estimated value of median recurrence-free survival was much better compared to that from previous studies (47.1 months over 12 to 21 months). At the follow-up period of 32.1 months, the median overall survival was not reached, with significantly reduced recurrence rate (30.3%). This study highlighted that a combination of ICIs and a peptide-based vaccine could provide enhanced immune activity with a promising survival rate for high-risk advanced melanoma.

The efficacy and safety of combined cancer vaccine and ipilimumab were evaluated in one of the biggest phase III trials for CTLA-4 checkpoint blockade, which enrolled patients who had previously been treated for malignant melanoma. This trial was a randomized and double-blinded study that recruited 676 patients with stage III to IV unresectable melanoma [142]. Among the enrolled patients, 403 patients were randomly received ipilimumab (3 mg/kg) in combination with a vaccine containing a monomer antigen (gp100 HLA-A:0201), whereas 137 patients received ipilimumab alone (3 mg/kg) and the remaining 136 patients received the gp100 HLA-A:0201 vaccine alone. In this study, ipilimumab with or without a gp100 peptide vaccine improved survival compared to gp100 alone. With these successful outcomes, ipilimumab was approved by FDA in 2011 as a treatment for patients with inoperable or metastatic melanoma.

A combination of ipilimumab with another type of peptide-based vaccine (MART-1/gp100/tyrosinase with Montanide ISA 51 VG) as an adjuvant was evaluated in high-risk resection of stage IIIC to IV melanoma. In the first single-arm trial, 19 patients were treated with ipilimumab at three different doses together with the multipeptide [143]. Compared to previous reports, this study reported higher response rates to specific peptides (47%) and lower disease recurrence rates in patients with autoimmune diseases. In another phase II trial, randomized treatment with ipilimumab at an extended dose (3 or 10 mg/kg) was given to 75 registered patients every 6-8 weeks along with subcutaneous delivery of the peptide-based vaccine [144]. Vaccination induced a gradual increase in the frequency of activated T cells, but immune response to the specific multipeptide was only detected in 25% of patients. Furthermore, 37% of patients demonstrated response toward MART-1 and reactivity to gp100 correlated with time to relapse. Adverse events caused by the combination therapy were mostly easy to manage and there was no treatment-related mortality. Symptoms that were frequently associated with grade 3 or 4 adverse events included diarrhea, colitis, and hypopituitarism, which were found in 29% of patients. Systemic steroid was successfully tapered off and patients were fully recovered within 3 months.

In addition to the vaccines that target TAAs, a therapeutic HPV-16 SLP vaccine has recently been developed to amplify HPV-specific T cell responses and to improve clinical response rate led by PD-1 inhibitor in patients with HPV-16-positive oropharyngeal squamous cell carcinoma [113]. In a similar way, a DNA vaccine that is designed to target HPV-16/18 E6 and E7 proteins robustly induced HPV-16/18-specific immune response in patients with HPV-associated cervical intraepithelial neoplasia [145] and head and neck cancer [146]. One patient who previously received anti-PD-1 antibody showed complete tumor regression (NCT03162224). Similar strategies were also tested in patients with HPV-associated cervix cancer [147], showing encouraging results.

NeoVax is a peptide-based, personalized neoantigen-based vaccine tested in a phase 1 trial with four patients with previous history of high-risk stage III and two patients with stage IV melanoma after initial surgical resection [148]. The NeoVax vaccine is constructed with 20 different long peptides (15–30 mers) and administered with adjuvant poly ICLC (TLR3 agonist consisting of carboxymethyl cellulose, polyinosine-polycytidyl acid and poly l-lysine double-stranded RNA). Neoantigen-specific CD4^+^ and CD8^+^ T cells became detectable following vaccination, and more robust immune response was observed mainly in CD4^+^ T cells. Transcriptional analysis revealed that, after vaccination, neoantigen-specific CD4^+^ T cells exhibited gene expression profiles of T helper 1 (Th1), effector and memory programs. Four patients suffering from stage III disease remained disease-free after vaccination all the way to a median follow-up period of 25 months (range 20–32 months). At stage IV disease, two patients experienced a relapse of disease several months after their final vaccination and immediate treatment of pembrolizumab resulted in complete regression of metastatic tumors as well as enhanced antitumor T-cell responses in these patients. Taken together, these findings suggest that combination therapies have the potential to further enhance the vaccine-driven T cell responses.

In the phase 1b NT-001 trial, several patients with advanced-stage melanoma, NSCLC, or urothelial cancer were enrolled and treated with a modified long-peptide vaccine constructed with poly ICLC (NEO-PV-01) in combination with nivolumab [89]. The monotherapy of nivolumab was administered to patients during vaccination and after vaccination as well as in the course of vaccine manufacture at the individual patient level. Using an ex vivo assay with peripheral blood samples, it was confirmed that all patients who were vaccinated showed neoantigen-specific T cell responses. TCRs specific for neoantigens were detected in two different patients with melanoma. Of the tumor sample from one patient who had a post-treatment stable disease status, the neoantigen-specific TCR was detected, which illustrates the vaccine-induced trafficking of neoantigen-specific T cells into the metastatic tumors. It remains to be determined whether the clinical benefit observed after vaccination was achieved solely by the use of vaccine or if it was due to the nivolumab treatment. Moreover, the trial was performed under a non-randomized study design. Interestingly, epitope spreading induced by vaccination promoted T cell responses to neoantigens that were irrelevant of the contents of the vaccine. This epitope spreading was strongly correlated with limited disease progression at 6 months after initial treatment in the patients with urothelial cancer and at 9 months in the patients with NSCLC and melanoma (intergroup *p* = 0.03), all of which improved progression-free survival of patients in these three different groups (HR 0.23, 95% CI 0.06–0.83; *p* = 0.01). These findings highlight the existence of a clinical benefit that is induced by the vaccines. The NEO-PV-01 was well tolerated when administered in combination with nivolumab and exhibited mild injection site reactions (52% of patients) and short-term flu-like manifestations (35% of patients).

In the case of lipoplex vaccine, RO7198457 is a personalized neoantigen-based RNA vaccine that encodes up to 20 neoantigens. In a phase Ib study, the RNA-lipoplex vaccine in combination with anti-PD-L1 antibody, atezolizumab, was administered to 132 enrolled patients with advanced stage solid tumors (NCT03289962). Among the participants, 77% of patients showed circulating T cell responses to an average of 2.6 neoantigens ex vivo. The frequency of CD8^+^ T cells specific for vaccine-induced neoantigens became detectable in peripheral blood (~5%). These neoantigen-specific CD8^+^ T cells were characterized by effector-memory phenotype with elevated expression of PD-1. Although TCRs specific for the vaccine-induced neoantigens were observed post-vaccination, they were undetectable in the tumor specimens before vaccination. Only about 7% of patients (28/132) with various advanced-stage solid tumors showed antitumor activity induced by RO7198475 in combination with atezolizumab. Adverse events related to the treatment were generally systemic symptoms such as low-grade cytokine release syndrome, infusion-related reactions, or flu-like symptoms.

In the case of neoadjuvant treatment, ICI impressively ameliorated pathological responses and induced generation of neoantigen-specific T cells in the peripheral blood and tumors [149]. For example, neoadjuvant treatment coupled with ICIs in the presence or absence of induction chemotherapy followed by resection of tumor resulted in major or complete pathological reaction from 37% up to 85% in non-small cell lung cancer [150], Merkel cell carcinoma [151], triple-negative breast cancer [152], and colon cancer with or without mismatch repair deficiency [153]. Likewise, a combination of neoadjuvant treatment and PROSTVAC vaccine has been reported to activate T cells in the periphery as well as leading to their infiltration into the TME of prostate cancer in patients [154]. Applying maintenance vaccination following tumor resection may allow long-term clinical response as well as preventing recurrence. Overall, carefully designing a suitable combination of treatments optimized for each individual patient and each tumor type will open the gate to future clinical advances in the development of cancer vaccines. Table 2 provides an overview of current combinatory strategies with ICIs and vaccines.

## 6. Conclusions and Perspectives

Enormous efforts have been dedicated to overcoming the failures of developing cancer vaccines in recent decades. As a result, significant progress has been made in improving existing strategies for cancer immunotherapy as well as in establishing new cancer vaccine platforms and methods for discovering target antigens. As further work is still required to accomplish the ultimate goal of establishing safe and efficient personalized cancer treatment, there are currently a large number of continuous and future trials of therapeutic cancer vaccines aiming to build up confidence in the application of these strategies. Importantly, combinatorial treatment involving newly designed ICIs or the recently identified co-stimulatory pathways, as well as other immunomodulatory approaches, along with the use of therapeutic cancer vaccines, will allow us to look forward to a successful clinical outcome. In the process of developing personalized cancer immunotherapy, a variety of genomic and protein biomarkers that can accurately predict for corresponding responses through molecular profiling of tumors and host cells using next-generation sequencing is expected to support critical decisions and improve clinical outcomes. In summary, cancer vaccines may become the next favored combination coupled with various strategies, which will introduce a newly designed platform that easily combines most of the existing therapies with minimized toxicity and impressive efficacy. In the near future, various therapies with different but complementary antitumor efficacies may be selected as optimal combination partners.

## Figures and Tables

**Table 1:** Classification of tumor antigens.

Categories	Tumor-Associated Antigens			Tumor-Specific Antigens		
Target types	Differentiation antigens	Overexpressed antigens	Cancer testis antigens	Oncoviral antigens	Private neoantigens	Shared neoantigens
Description	Antigens expressed during tissue differentiation	Antigen overexpressed on tumor cells compared to normal cells	Antigens limitedly expressed on testes, fetal ovaries, and trophoblast	Antigen expressed on cells infected with oncovirus	Antigens resulting from somatic mutation of uniquely mutated gens	Antigens resulting from somatic mutation of recurrently mutated genes
Tumor specificity	Variable	Variable	Good	Ideal	Ideal	Ideal
Central tolerance	High	High	Low	None	None	None
Prevalence in multiple patients	High	High	High	High	Low	High
Examples	Melan A, CD19	HER2, TROP2	MAGE-A3, NY-ESO-1	EBV LMP, HPV E6/E7	Numerous	KRAS, p53

**Table 2:** Clinical evidence for combining vaccines and ICIs.

Vaccine	Combined ICIs	Patient Population	Phase	Enrolled Patients	Main Outcomes	Clinical Trial Identifier
T-VEC	Pembrolizumab	Unresectable stage IIIB-IVM1c melanoma	Ib	n = 21	Efficacy: CR 43%, 4-year PFS rate 56%, 4-year OS: 71% Safety: Well tolerated, with most common AEs being fatigue, chills, and pyrexia	NCT02263508
	Pembrolizumab	Unresectable stage IIIB-IVM1c melanoma	III	n = 713	Ongoing	NCT02263508
	Pembrolizumab	Recurrent or metastatic HNSCC	Ib	n = 36	Confirmed PR in 5 pts (13.9%), PFS and OS were 3.0 months [95%CI, 2.0–5.8] and 5.8 months (95% Cl, 2.9–11.4), respectively. One DLT of T-VEC-related was fatal arterial hemorrhage. Besides the DLT, there were no treatment-related fatal AEs.	NCT02626000
Multipeptide vaccine (MART-1/NY-ESO-1/gp100 with montanide ISA 51 VG)	Nivolumab	Unresectable stage III-IV melanoma	I	n = 90	RR for both ipilimumab-refractory and -naive pts was 25%. DOR was not reached at a median of 8.1 months of follow-up. Nivolumab with vaccine was well tolerated and safe at all doses.	NCT01176461
	Nivolumab	Resected stage IIIc-IV melanoma	I	n = 33	Estimated RFS was 47.1 months, extremely beneficial compared with historical RFS (12–21 months) Five G3 TRAEs include hypokalemia (n = 1), rash (n = 1), enteritis (n = 1), and colitis (n = 2).	NCT01176474
gp100 HLA-A:0201 vaccine	Ipilimumab	Unresectable stage III-IV melanoma	III	n = 676	OS was 10.0 months among pts receiving ipilimumab plus gp100, as compared with 6.4 months among pts receiving gp100 alone (hazard ratio for death, 0.68; *p* < 0.001). G3/4 irAEs occurred in 10% to 15% of pts treated with ipilimumab and in 3% treated with gp100 alone.	NCT00094653
Multipeptide vaccine (MART-1/gp100/Tyrosinase with Montanide ISA 51 VG)	Ipilimumab	Resected stage IIIc-IV melanoma	I	n = 19	RR to specific peptides (47%) was higher than previous reports, and disease relapse rate was lower in patients with autoimmunity.	NCT00025181
	Ipilimumab	Resected stage IIIc-IV melanoma	II	n = 75	Autoimmune evidence positively correlating with improved RFS was observed in 37% of patients, but the combination failed to generate additional benefits. Frequently occurring G3/4 AEs were diarrhea, colitis, and hypopituitarism, which occurred in 29% of patients.	NCT00084656
SLP HPV-16 vaccine *ISA101*	Nivolumab	Unresectable HPV-positive cancer (oropharyngeal [n = 22], anal cancer [n = 1], and cervical cancer [n = 1])	II	n = 24	ORR was 33% (8 patients; 90% CI, 19–50%), DOR was 10.3 months (95% CI, 10.3 months to inestimable). 5 of 8 pts remain in response. PFS was 2.7 months (95% CI, 2.5–9.4 months). OS was 17.5 months (95% CI, 17.5 months to inestimable). G3/4 toxicity occurred in 2 pts (asymptomatic G3 transaminase level elevation (n = 1) and G4 lipase elevation (n = 1)), requiring discontinuation of nivolumab therapy.	NCT02426892
MEDI0457 (INO-3112) targeting the HPV-16/18 E6, E7 proteins	Durvalumab	HPV-associated recurrent and/or metastatic HNSCC	Ib/IIa	Recruiting	Ongoing	NCT03162224
GX-188E targeting the HPV-16/18 E6, E7 proteins	Pembrolizumab	Advanced, non-resectable HPV-positive cervical cancer	II	n = 36	At 24 wks, 11 (42%; 95% CI 23–63) of 26 pts achieved an OR; 4 (15%) had a CR and 7 (27%) had a PR. 16 (44%) of 36 pts had TRAEs of any grade and four (11%) had G3/4 TRAEs including G3 increased aspartate aminotransferase, syncope, pericardial effusion, and hyperkalemia, and G4 increased alanine aminotransferase.	NCT03444376
Poly-ICLC (NeoVax)	Pembrolizumab	Resected high-risk stage III/IV melanoma	I/Ib	n = 6	4 pts with stage III disease remained disease-free at a median follow-up duration of 25 months (range 20–32 months) and 2 pts with stage IV disease had disease recurrence within a few months after the last vaccination and subsequently received pembrolizumab.	NCT01970358
Poly-ICLC (NEO-PV-01)	Nivolumab	Advanced-stage melanoma (34),NSCLC (27), and urothelial cancer (21)	Ib	n = 82	Ongoing	NCT02897765
Personalized RNA-lipoplex neoantigen-based vaccine (RO7198457)	Atezolizumab	Advanced stagesolid tumors	Ib	n = 132	The antitumor activity of RO7198475 in combination with atezolizumab was observed in 28 pts (7%). TRAEs were predominantly systemic (low-grade cytokine release syndrome, infusion-related reactions, or flu-like symptoms.	NCT03289962

T-VEC, talimogen laherparepvec; CR, complete remission; PFS, progression-free survival; AE, adverse event; OS, overall survival; HNSCC, head and neck squamous carcinoma; CI, confidence interval; PR, partial response; DLT, dose limiting toxicity; RR, response rate; DOR, duration of response; TRAEs, treatment related adverse events; HLA, human leucocyte antigen; RFS, relapse free survival; irAEs, immune-related adverse events; pt, patient; SLP, synthetic long peptide; HPV, human papilloma virus; ORR, objective response rate; wks, weeks; OR, overall response.
